# The future of General Movement Assessment: The role of computer vision and machine learning – A scoping review

**DOI:** 10.1016/j.ridd.2021.103854

**Published:** 2021-03

**Authors:** Nelson Silva, Dajie Zhang, Tomas Kulvicius, Alexander Gail, Carla Barreiros, Stefanie Lindstaedt, Marc Kraft, Sven Bölte, Luise Poustka, Karin Nielsen-Saines, Florentin Wörgötter, Christa Einspieler, Peter B. Marschik

**Affiliations:** a iDN – Interdisciplinary Developmental Neuroscience, Division of Phoniatrics, Medical University of Graz, Graz, Austria; b Know-Center GmbH, Graz, Austria; c Child and Adolescent Psychiatry and Psychotherapy, University Medical Center Göttingen, Göttingen, Germany; d Leibniz-ScienceCampus Primate Cognition, Göttingen, Germany; e Department for Computational Neuroscience, Third Institute of Physics-Biophysics, Georg-August-University of Göttingen, Göttingen, Germany; f German Primate Center – Leibniz Institute for Primate Research, Göttingen, Germany; g Institute of Interactive Systems and Data Science, Graz University of Technology, Graz, Austria; h Department of Medical Engineering, Technical University Berlin, Berlin, Germany; i Center of Neurodevelopmental Disorders (KIND), Centre for Psychiatry Research, Department of Women’s and Children’s Health, Karolinska Institutet, Stockholm, Sweden; j Child and Adolescent Psychiatry, Stockholm Health Care Services, Region Stockholm, Stockholm, Sweden; k Curtin Autism Research Group, School of Occupational Therapy, Social Work and Speech Pathology, Curtin University, Perth, Western Australia, Australia; l Division of Pediatric Infectious Diseases, David Geffen UCLA School of Medicine, USA; m Institute of Physics, Department for Computational Neuroscience at the Bernstein Center Göttingen, Georg-August-University of Göttingen, Göttingen, Germany

**Keywords:** Augmented general movement assessment, Automation, Cerebral palsy, Computer vision, Deep learning, Developmental disorder, Early detection, General movements, Infancy, Machine learning, Neurodevelopment, Pose estimation

## Abstract

**Background:**

The clinical and scientific value of Prechtl general movement assessment (GMA) has been increasingly recognised, which has extended beyond the detection of cerebral palsy throughout the years. With advancing computer science, a surging interest in developing automated GMA emerges.

**Aims:**

In this scoping review, we focused on video-based approaches, since it remains authentic to the non-intrusive principle of the classic GMA. Specifically, we aimed to provide an overview of recent video-based approaches targeting GMs; identify their techniques for movement detection and classification; examine if the technological solutions conform to the fundamental concepts of GMA; and discuss the challenges of developing automated GMA.

**Methods and procedures:**

We performed a systematic search for computer vision-based studies on GMs.

**Outcomes and results:**

We identified 40 peer-reviewed articles, most (n = 30) were published between 2017 and 2020. A wide variety of sensing, tracking, detection, and classification tools for computer vision-based GMA were found. Only a small portion of these studies applied deep learning approaches. A comprehensive comparison between data acquisition and sensing setups across the reviewed studies, highlighting limitations and advantages of each modality in performing automated GMA is provided.

**Conclusions and implications:**

A “method-of-choice” for automated GMA does not exist. Besides creating large datasets, understanding the fundamental concepts and prerequisites of GMA is necessary for developing automated solutions. Future research shall look beyond the narrow field of detecting cerebral palsy and open up to the full potential of applying GMA to enable an even broader application.

## Highlights

• A wide variety of tracking and detection tools for computer vision-based GMA exist.
• A “method-of-choice” for automated GMA does not yet exist.
• Large expert-annotated valid datasets are urgently needed.
• The prerequisites of classic GMA is indispensable for developing automated solutions.
• A future augmented GMA shall combine human expertise with computerised tools.

## What this paper adds?

• An overview of computer vision-based approaches in the study of general movements is provided.
• The advantages, limitations, and future directions of vison-based approaches in performing automated general movement assessment (GMA) are discussed.
• Prospects of computer-driven GMA are discussed. The necessity of understanding the nature of general movements and GMA while developing automated solutions is highlighted.
• It is suggested that future research shall look beyond the narrow field of detecting cerebral palsy and open up to the potential of applying GMA to identify more general disintegrity of the developing nervous system in early infancy.

## 1 Introduction

Early detection of developmental disorders of various aetiologies, which are usually diagnosed during toddler-years or older, is a major challenge to clinicians and scientists across disciplines. Over the years, this field has become increasingly complex and has incorporated developmental, clinical, as well as technical perspectives. Besides the classic biomarker approaches targeting earlier identification of such *late detected developmental disorders* (LDDDs), the assessment of neurofunctional or behavioural biomarkers has caught increasing attention (e.g., Varcin & Nelson, 2016; Marschik, Einspieler, Sigafoos, Enzinger, & Bölte, 2016; Marschik et al., 2017; Peyton & Einspieler, 2018). Research in different behavioural domains from early life and onwards has adopted both retrospective and prospective paradigms, such as the retrospective work on Rett syndrome (e.g., Einspieler & Marschik, 2019), or the ever-growing field of prospective siblings studies on autism spectrum disorders (e.g., Ali, Charman, Johnson, Jones, & Team, 2020; Bölte et al., 2016; McDonald et al., 2020; Murphy & Spooren, 2012; Ozonoff et al., 2015; Shephard et al., 2019)

In this scoping review, we address one specific behavioural domain, the developing motor functions in the first few months of life. We focus on the subdomain of spontaneous *general movements* (GMs) and aim to recapitulate current computer vision-based studies on tracking and detection of GMs.

First operationalised by Heinz Prechtl and colleagues (e.g., Einspieler, Marschik, & Prechtl, 2008; Prechtl, 1990; Prechtl et al., 1997), the assessment of GMs has opened a unique window for scientists and clinicians to sight with their bare eyes the integrity of the young developing nervous system. Our interdisciplinary developmental neuroscience lab and the systemic ethology and development research lab, originated and founded by Heinz Prechtl and Christa Einspieler, inherit the long tradition and rich experience of studying GMs and bear the mission to extend the knowledge of GMs. Maintaining the high standard of the Prechtl general movement assessment (GMA), it is our vision to translate the classic GM field, the prediction of cerebral palsy (CP), to broader applications, incorporating innovative routes and wider perspectives.

GMs are but a part of the spontaneous movement repertoire (i.e., not induced by any external stimulus) and are present from early foetal life towards the end of the first half-year postterm. GMs involve the entire body, hence the term *general* movements. GMs are variable sequences of movements of the arm, leg, neck, and trunk with changing intensity, force, and speed (e.g., Einspieler, Marschik et al., 2008; Einspieler & Prechtl, 2005). A sequence of GMs waxes and wanes gradually, involving fluent and elegant rotations along the limbs’ axis and slight changes in the movement direction. GMs are complex in appearance, and importantly, variable. When the developing nervous system is impaired, GMs lose complexity; their smooth and variable character alters and becomes monotonous, abrupt, or disorganised. Importantly, GMs present distinct age-specific patterns during the pre-term and term periods, and at 3−5 months of age. While at term age and shortly after, the writhing movements (WMs) dominate, the fidgety movements (FMs) gradually set in between 6–8 weeks, become pronounced at 12–16 weeks, and vanish around 20 weeks of postterm age (PTA). The quality of GMs can be examined by the Prechtl GMA, one of the most sensitive and reliable diagnostic tools for the prediction of cerebral palsy (e.g., Kwong, Fitzgerald, Doyle, Cheong, & Spittle, 2018; Novak et al., 2017; Prechtl et al., 1997). Quality of GMs is defined into age-specific normal vs abnormal categories. Abnormal GM patterns during the writhing movement period include: *poor repertoire*, *cramped-synchronized*, or *chaotic* movements; and during the fidgety movement period: *abnormal* or *absent* fidgety movement patterns. Especially, normal FMs suggest normal neurological development while the absence of FMs at 3−5 months PTA is the most sensitive and specific indicator of later neurological impairments, such as cerebral palsy (e.g., Bosanquet, Copeland, Ware, & Boyd, 2013; Einspieler, Bos et al., 2019; Einspieler, Utsch et al., 2019; Einspieler, Peharz, & Marschik, 2016; Einspieler & Prechtl, 2005; Kwong et al., 2018; Prechtl et al., 1997).

Initially a powerful predictor of CP, general movements have been studied worldwide in a multitude of neurodevelopmental and genetic disorders (e.g., Herrero et al., 2017; Romeo et al., 2008; Tomantschger et al., 2018). Accumulating evidence reveals elevated occurrences of aberrant GMs in infants later diagnosed with LDDDs, e.g., autism spectrum disorder, Rett syndrome (e.g., Einspieler et al., 2014; Einspieler & Marschik, 2019; Zappella et al., 2015), or a range of early-identifiable disorders such as Down syndrome (e.g., Herrero et al., 2017) and Cornelia de Lange syndrome (e.g., Marschik, Soloveichick, Windpassinger, & Einspieler, 2015). Abnormal GMs are also present in infants born to mothers with viral infections like HIV or Zika that affect the central nervous system (e.g., Brasil et al., 2016; Einspieler, Utsch et al., 2019; Einspieler & Marschik, 2020a, 2020b; Palchik, Einspieler, Evstafeyeva, Talisa, & Marschik, 2013; Soares-Marangoni et al., 2019). The significance of GMs in early brain development in general and, consequentially, its long-term relevance for the later development of cognitive, speech-language, and motor functions has been increasingly recognised (e.g., Einspieler, Bos, Libertus, & Marschik, 2016; Einspieler, Peharz et al., 2016; Grunewaldt et al., 2014; Salavati et al., 2017). Although abnormal GMs, especially the absence of FMs during 3−5 months, do not point to a specific disorder, they flag high risks for future neurological impairments. If GMA could be manualised in daily clinical routines, it would support the earlier identification of LDDDs and other neurodevelopmental impairments. Infants identified with abnormal GMs would be monitored more closely, and could thus, be referred sooner for specific diagnostic evaluations and benefit earlier from interventions (e.g., Peyton & Einspieler, 2018; Zang et al., 2016).

As GMA requires only 3−5 minutes observation of an infant’s spontaneous movement (i.e., the infant needs not to be touched by the assessor), it is an evaluation far easier to be carried out than most assessments for neurological development. Hence GMA is suitable for daily clinical applications, particularly in low-resource settings. Being entirely non-intrusive, GMA is widely accepted by caregivers with divergent social and cultural backgrounds (e.g., Burger & Louw, 2009; Soleimani, Teymouri, & Biglarian, 2013; Tomantschger et al., 2018).

However, GMA can only be performed by certified assessors. Acquiring specific high-quality training is a prerequisite for a GMA assessor, and regular practices and recalibrations are indispensable. This is one reason why GMA has not yet been established universally in the daily clinical routines. Although interrater reliability of GMA has proven to be excellent across various studies at different sites (e.g., Einspieler & Prechtl, 2005; Kwong et al., 2018; Valle, Støen, Sæther, Jensenius, & Adde, 2015; Yuge et al., 2011), assessor skills surely vary from individual to individual and can be influenced by adverse human or environmental factors. So much as the clinical and scientific credit of GMA has been acknowledged, we need complementing avenues to scale up this valuable tool, where modern technology may be able to play a more decisive role. Indeed, in the past two decades, a boom of technological approaches aiming at automated or technology-assisted GMA have surfaced. These efforts range from mobile-app-based recording tools, e.g., the Baby Moves (Spittle et al., 2016) and the GMApp (Marschik, Pokorny, et al., 2017), to automated pose estimation through sensor-based or markerless approaches (e.g., Irshad, Nisar, Gouverneur, Rapp, & Grzegorzek, 2020; Marcroft, Khan, Embleton, Trenell, & Plötz, 2015; Marschik et al., 2017).

In this paper, we provide an in-depth analysis of the most recent technology-driven studies on GMs. We focus on video-based approaches only, since GMA is in origin a visual-based method. Advanced computer vision technology remains authentic to the non-intrusive character of the classic GMA, allowing automated analyses of the infant’s spontaneous movements, which is not influenced by the use of wearable sensors and other devices (for recent overviews on diverse sensors targeting GMs, please see Hyde et al., 2019; Irshad et al., 2020; Marcroft et al., 2015). Different from previous reviews, we examine in particular if the existent technological attempts targeting automated GMA are in accordance with the fundamental concepts underlying this unique clinical tool. Specifically, we aim to: (1) provide an overview of available video-based approaches targeting GMs; (2) identify their techniques for movement detection, tracking, data pre-processing, and classification; and most importantly, (3) discuss from both, the conceptual and the technological perspectives, the major challenges, as well as advantages of incorporating automated visual-based approaches into classic GMA to enable an even broader application in daily clinical routines.

## 2 Materials and methods

### 2.1 Search methods

**Fig. 1:**
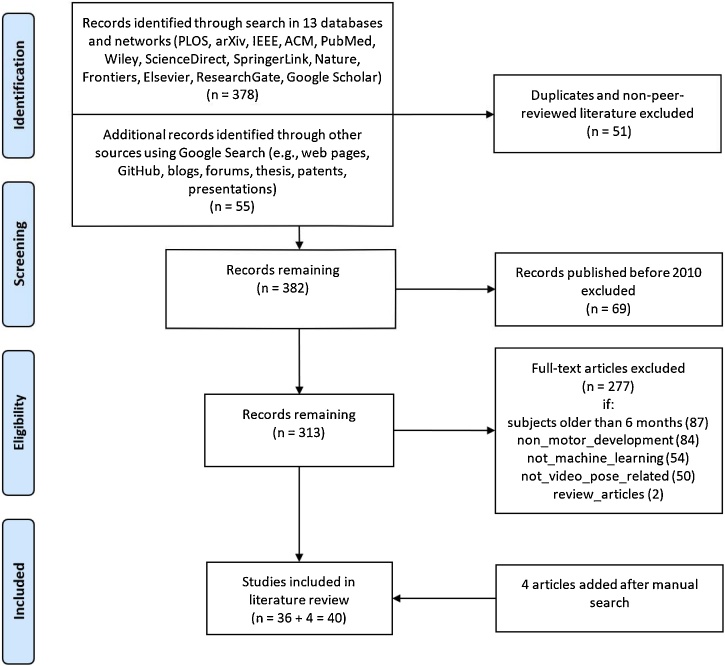
Literature search and screening procedure.

A search with thirteen well-known databases and research networks (Fig. 1) was carried out in September 2020. Fig. 1 summarises the complete search and screening procedure. In addition to the thirteen different sources, we also searched in Google, including personal webpages, blogs, forums, thesis, patents, and performed ancestral research of published papers to collect additional studies.

Following our aims, we defined three core categories of interests (COIs) for the search process: general movements, machine learning, and computer vision. Search terms of each COI are presented in Table 1. Studies published in English and found to be related to the three COIs were all collected and organised first via Zotero (Zotero (about), 2020). All the records were subsequently exported from Zotero to the visualisation tool SurVis (Beck, Koch, & Weiskopf, 2016) for automatic analysis of publication dates, keywords, authors, and topic clusters. We applied text analysis (using R) to examine the full texts (e.g., leading journals, top keywords). Our search concentrated on technological approaches and studies of infants, covering applications on both the automated analysis of movements and the early detection/prediction of developmental disorders. The search resulted in a total of 433 relevant records. In the following step, we screened these 433 records.

**Table 1:** Search categories and terms.

**Categories**	**Search Terms**
**General** **Movements**	baby OR child* OR infan* OR newborn OR *term OR neonatal OR abnormal OR anomaly OR atypical OR disorder OR risk OR sign* OR typical OR diagnos* OR analys* OR early OR assessment OR behavio* OR *marker OR cerebral palsy OR development* OR fidgety OR body OR gma OR gm OR outcome OR general movement* OR motor AND
**Machine** **Learning**	accuracy OR adaptive OR advanced OR auto OR biosensor OR classification OR detect* OR learn* predict* OR recognition OR recommend* OR sens* OR neuro* OR algorithm* OR deep OR model* OR machine AND
**Computer** **Vision**	2d OR 3d OR action OR activity OR classifier OR estimat* OR framework OR human OR intelligen* OR motion OR pose OR predict OR tracking OR video

### 2.2 Screening

First, all duplicates and non-peer-reviewed articles were deleted. Second, articles published before 2010 were excluded to focus on the significant technological advancements during the past decade. Third, we removed studies of older infants (participants were on average 6 months of age or older); studies not targeting GMs; studies that did not apply machine learning; or studies that did not use video-based techniques.

## 3 Results

According to our search and screening procedures, we identified 40 peer-reviewed articles, 10 being conference contributions. All of these studies provided in-depth technical and algorithmic details on infant movement analysis and applied automated video-based approaches with machine and deep learning techniques (Table 2). Most studies (n = 30) were published between 2017 and 2020, indicating a strong interest in, and a boom of, video-based approaches addressing GMA in the recent years.

**Table 2:** Video-based technological approaches for studying GMs.




* Articles are first arranged in descending order of the publication year, followed by ascending order of the last name of the first author. Studies with an inherent connection, i.e., leading authors are identical or worked jointly, are stacked together and shaded with the same background colour, also ordered first by the publication year and then by the last name of the first author.

** Studies in which the ages of the participants fell (partly) beyond the appropriate range according to the standard GMA (Einspieler et al., 2014), or the age range was (partly) missing.

**Key of Terms.**

*Generic*: ASD – Autism Spectrum Disorder; CP – Cerebral Palsy; CS – Cramped Synchronised; FM – Fidgety Movements; GA – Gestational Age; GMS – General Movements; GMA – General Movement Assessment; NA – Not Applicable; NR – Not Reported; PTA – Postterm age. PR – Poor Repertoire; WM – Writhing Movements.

*Techniques and Models*: A_SD_ – Acceleration Standard Deviation; CIMA – Computer-based Infant Movement Assessment; CPP – Cerebral Palsy Predictor; C_SD_ – Standard Deviation of the Center of Motion; FFT – Fast Fourier Transformation; HOJD2D – Histograms of Joint Displacement 2D; HOJO2D – Histograms of Joint Orientation 2D; ICC – Intraclass Correlation Coefficient; LDA – Linear Discriminant Analysis; LDOF – Large Displacement Optical Flow; LLGMN – Log-linearised Gaussian Mixture; LR – Logistic Regression; MEMD – Multivariate Empirical Mode Decomposition; MRF – Multi-label Markov Random Field; NGBS – Naive Gaussian Bayesian Surprise; PCKh 2.0 – Percentage of Correct Keypoints in Relation to Head Segment Length (two times the head segment length); QMEAN – Quantity of Motion Mean; Qmedian – Quantity of Motion Median; Q_SD_ – Quantity of Motion Standard Deviation; RBF – Radial Basis Function Kernel; RF – Random Forests; RPSR – Robust Point Set Registration; SMIL – 3D Skinned Multi-Infant Linear (Based on SMPL Model for Adults); SMPL – Skinned Multi-Person Linear Model; SVM – Support Vector Machine; V_SD_ – Standard Velocity Deviation.

Table 2 provides a detailed overview of the various studies targeting automated GMA, summarising their approaches and techniques on infant movement detection, tracking, and classification. Table 3 provides a comprehensive comparison between data acquisition and sensing setups across the reviewed studies, highlighting limitations and advantages of each modality in performing automated GMA.

**Table 3:** Challenges, limitations, and future directions of different computer vision sensing and data processing approaches.

	**Current approaches / Problems**	**Future directions / Improvements/ Challenges**
**Sensors**	**Current approaches** Mostly 2D single cameras **Problems** Only 2D information Occlusions	**Future directions** Multiple 2D cameras 3D (depth) sensors Pressure mat sensors **Improvements** 3D information Less occlusions More information due to multi-sensory integration
**Data**	**Current approaches** Small datasets **Problems** Not enough data to employ deep learning methods Not publicly available – no benchmarking possible Incorrect or incomplete data in some cases, e.g., inaccurate outcome labelling due to lack of longitudinal studies, the inclusion of incorrect age-specificity cases, use of low-inter-rater agreement or small rater-group or lack of experienced raters in data labelling, disorders or gender misrepresentation	**Future directions** Collect more data Make it publicly available Make use of home videos Employ DL methods Make use of transfer learning (e.g., Tan et al., 2018) **Challenges** Need to solve anonymisation issue (automated techniques for face detection and replacement can be applied) Development of methods which can cope with different light conditions, resolution, frame rate
**Body areas of interest**	**Current approaches** Mostly movement of arms, legs, head **Problems** Incomplete information of full-body movement	**Future directions** Hand, fingers, feet Eye movement data Mimic **Challenges** Integration and analysis of multimodal information
**Motion tracking**	**Current approaches** Mostly in 2D space **Problems** Only 2D information	**Future directions** Full-body tracking in 3D using well-established methods in DL (e.g., DeepLabCut and OpenPose frameworks) **Challenges** DL methods need to be adapted to infants
**Motion encoding**	**Current approaches** Conventional features based on: displacement, distance, velocity, acceleration, speed, and time **Problems** Only 2D features	**Future directions** Motion encoding using well-established methods from robotics: Dynamic Movement Primitives (e.g., Ijspeert, Nakanishi, Hoffmann, Pastor, & Schaal, 2013), Gaussian Mixture Models (e.g., Calinon, 2016; Khansari-Zadeh & Billard, 2011), Probabilistic Movement Primitives (Paraschos, Daniel, Peters, & Neumann, 2018) Learn features from expert knowledge during observation (e.g., Silva et al., 2018, 2019) **Improvements** 3D features New motion encoding and features
**Classification algorithms**	**Current approaches** Conventional ML methods, e.g., SVM, Decision Trees, Neural Networks, Hidden Markov Models Supervised learning without feedback during learning	**Future directions** Employ ANN, DL Employ Interactive Machine Learning (learning with feedback) **Improvements** Better models with more accurate predictions **Challenges** More data is needed

In Table 2, we split the studies into two generic groups: conventional machine learning models (CML, n = 35; e.g., SVM, random forest) and deep learning models (DL, n = 5; e.g., CNN, LSTM). In regards to tracking and detection techniques, 12 CML studies and 4 DL studies applied pose estimation (with OpenPose or a custom pose implementation). The other 24 CML studies applied diverse tracking and detection methods.

Multiple motion-related techniques were exploited, such as Optical Flow, i.e., a technique for tracking the motion of an infant across multiple frames to estimate the velocity of body parts and predict the position of each body part in the next frame, or a Particle Filter used as a technique for localisation and mapping in Optical Flow, or Graph-cut, a graph-based segmentation technique used before executing a Particle Filter.

As presented in Table 2, a variety of movement features were extracted, such as kinematic features (i.e., standard or customised features that define velocity and acceleration of points in a moving body); frequencies, amplitudes, and covariation of movement’ parameters (e.g., position, velocity, or acceleration); other spectral components (e.g., harmonics in periodic vibrations in moving body parts, used for FMs detection). Using pose estimation, Orlandi et al. (2018) developed a new set of time-related features to detect FMs. Moccia, Migliorelli, Carnielli, and Frontoni (2020), with a newly invented “*Pose Tool*”, calculated the standard deviation of joint angles over time by using visual indicators to represent such deviations. Cenci, Liciotti, Frontoni, Zingaretti, and Carnielli (2017) introduced a new movement state-vector to their model defining whether a targeted body part *is* or *is not* in motion by modelling the infant’s movement sequence as a series of transitional states using a Markov Chain (MC).

To categorise these features, diverse computational algorithms were used, such as KAZE, i.e., a multiscale 2D feature detection and description algorithm (Alcantarilla, Bartoli, & Davison, 2012), Large Displacement Optical Flow (LDOF), i.e., an integration of rich descriptors into a variational optical flow setting to detect small-fast moving body parts (J. M. Brox, 2011; T. Brox, Bruhn, Papenberg, & Weickert, 2004), Markov Random Fields (MRF), i.e., used to encode contextual constraints into the prior probability (Pal & Pal, 1993), and Random Spectral Regression (RSR), i.e., a human action recognition algorithm based on random spectral regression (Lin, Zhu, Fan, & Fan, 2011).

## 4 Discussion

Over the past decade, the significant clinical and scientific value of the Prechtl GMA has been increasingly recognised. Armed with the rapid advancing computer science, a surging interest in developing automated GMA prevails in the field. Among the identified studies directly devoted to automated vision-based GMA, the majority were published within the past five years, and more are coming day after day (e.g., Doroniewicz et al., 2020; Groos, Adde, Støen, Ramampiaro, & Ihlen, 2020). As a limitation, we targeted only the publications in English during the past decade. Some work in the field could hence have escaped our review. Still, this scoping review, which aims at mapping the key concepts underpinning the research area of vision-based GMA, reflects on the cutting-edge of the field. In this section, we discuss the current approaches addressing automated solutions of GMA from both conceptual and technological perspectives.

### 4.1 Conceptual considerations

Given the expanding interests in automated movement analysis, any attempt to develop computer-driven GMA requires a genuine understanding of the underlying concepts of the GMs and an intensified scientific sensitivity. GMA is by nature *gestalt*, the perception and interpretation of the infant’s entire movement pattern without emphasising isolated parts. By contrast, computer-based methods are built upon minute *features* to generate algorithms. Although the automated GMA aims at overall classification, it remains a critical question, if and how human gestalt perception can be appropriately emulated by artificial intelligence (AI)? To validate tech-driven GMA, not only the interpretation of the classes, but more importantly, the extracted features, especially those obtained with unsupervised machine learning techniques, are of great conceptual, theoretical, and clinical importance. Otherwise, we might end up with merely a handful of discrete labels while losing the essential scientific and clinical semantics of GMA. To this end, we would need a more open communication between GMA experts and computer scientists to ensure the validity of future computerised models.

Speaking of the fundamental concepts of GMA, GMs are a significant constituent of the young infants’ broad spontaneous movement repertoire and must be observed within the specific age span. As introduced at the beginning, infant movement patterns change dramatically during the very first months of life. Movements around term age are qualitatively different from the ones during the 3−5 month period, as these motor patterns mirror the developmental status of the nervous system at each respective age. Unfortunately, essential information on the participants’ characteristics (e.g., the gestational age) was frequently missing in the discussed studies (Table 2). Some studies, although technically related to automated GMA, sampled infants beyond the age at which the GMs could be observed (e.g., Ouss et al., 2018, 2020). This implies that the classification algorithms of these studies might have been built upon (at least partly) inappropriate inputs, and the prediction would then have little to do with GMA per se. Relatedly, the current motion-tracking libraries and frameworks are mostly based on models for tracking adult movements, which are inherently different from those of the young infants. There is a need for further exploration as to how and if these “large-body oriented” motion tracking frameworks could be adapted to track infants’ body parts and joints, as well as their motor specificities with suitable recording setups. *Infant-specific* models are needed in their own right to account for the subject’s age-specific anatomical and motor constraints (Hesse, Pujades et al., 2018; Hesse, Schroeder et al., 2018; Ihlen et al., 2020).

Needless to say, computational models can only make predictions based on the datasets they are trained on, no more and no less. The nature of the input for training a model inevitably determines the validity and quality of its output. While attempting to acquire data for creating algorithms for automated GMA, we face the following challenges:

#### 4.1.1 Sample attributes

Besides the age-specificity issue discussed above, we need to ask which high-risk groups or disorders are targeted (e.g., preterm-infants, who are at elevated risk for developing CP)? Is an adequate and appropriate control group (i.e., typically developing infants) included, which is important for all machine learning methods (Schmidt, Regan, Fahey, & Paplinski, 2019)? Is the sample representative for the targeted group and the sample size (number of participants and the amount of data from each infant) sufficient, so that the outcome is reliable and generalisable? We should not forget that GMs have a large, complex, and variable repertoire, bringing difficulties for machine learning approaches to acquire a representative dataset. For example, when relying on retrospective videos from infants with atypical development, due to the uncertain representativeness of the training datasets, it might be challenging to achieve high external validity when testing the created model on novel samples (Irshad et al., 2020).

#### 4.1.2 Sensing and recording setups

As previously mentioned, despite the type of camera setups (Table 3), the non-intrusive classic GMA requires a standard viewing perspective to observe the infant’s entire body. The infant is in supine position and untouched, moving free of any external stimulus, and should also be in an appropriate behavioural state (Einspieler et al., 2014). Otherwise, the movement pattern could be distorted. To maintain the non-intrusive character of the GMA, vision-based markerless approaches appear more favourable than the ones using wearable sensors, or attaching markers to the infant’s body. Although marker- and wearable sensor-based approaches have technical merits (see Irshad et al., 2020), it is yet to be examined whether these markers or sensors may interfere with the infants’ spontaneous movements, or whether the device-attaching procedures, usually time-consuming and during which the infant has to be touched or manipulated, could affect the infant’s consequential behavioural state (e.g., becoming fussy and distracted).

#### 4.1.3 Dataset annotation and segmentation

The quality of the annotation, being a key for the machine learning training dataset and the basis for classification, is largely neglected in the majority of the reviewed articles. In most cases, no information was provided on whether the dataset was annotated by certified GMA assessors, let alone the inter- and intra-rater reliability of the annotation by the GMA assessors. At the moment, no expert-annotated and validated public accessible large datasets are yet available for the purpose of scientific research. To realise automated GMA, creating such datasets might be challenging, partly due to complex confidentiality and privacy issues, which are however indispensable.

During the data annotation procedure, the duration of the video segments to be labelled is another puzzling issue. For machine learning methods, the shorter the movement duration, the less complex the model (i.e., less parameters), and thus, the shorter the time needed for training the model (assuming that shorter movement durations lead to smaller feature vectors). For human assessors, however, a 2−5 min observation is normally required by the GMA standard to evaluate the infant’s movement repertoire. It is yet to be explored, if human assessors are capable of annotating very short video clips confidently and reliably. More importantly, it is normal if a desired type of movements (targeted by the computer model), for example, the FMs, is absent for a short interval (e.g., 5 s) in a fully typically developing infant, who is in the fidgety movement age period. If the data annotation would be based, for example, on 20-second clips each, where both the fidgety movements (“1″) *and* non-fidgety movements (“0″) could occur, an annotation of either “0″ or “1″ for the respective 20 s could be inappropriate. That said, we need to find a compromise between a reasonable unit duration, appropriate feature encoding for machine learning algorithm and a minimum length of video for human assessors to be able to evaluate.

### 4.2 Technological considerations

From a technological perspective, a wide variety of sensing, tracking, detection, and classification tools for automated GMA based on computer vision are available (Table 2). Not only research approaches are heterogeneous, their datasets for training and testing across studies are also divergent. For this reason, a cross-study comparison on the model performance is almost impossible. Only a small portion of the existing studies applied deep learning approaches (DL, n = 5), which is likely to change in the near future. DL, being able to extract latent data features in an unsupervised way (e.g., using autoencoder architectures), is more suitable for handling massive datasets to achieve high performance. Efforts on creating larger validated datasets are needed and will allow further advancements in developing the DL models.

Given the various techniques applied, no current automated solution could yet defeat human GMA experts. Consequently, a fully automated GMA for the clinical practice seems rather elusive in the near future. To increase the performance of the technical approaches, on one hand, we need to better comprehend the underlying principles of the classic GMA, create larger annotated valid datasets, and revisit the capability and limitation of the existing approaches; on the other hand, we might need to develop novel strategies. For example, in addition to traditional methods to prevent overfitting such as training using early validation stop or utility of drop-out layers in DL, we could introduce additional regularisation methods (e.g., noise injection; Kukačka, Golkov, & Cremers, 2017) to the models to reduce overfitting and therefore increase their generalisation properties. It may be beneficial to transfer motion information acquired using DL approaches of different application domains to pose estimation of infants (e.g., Sim2Real; Doersch & Zisserman, 2019); or, to adopt interactive machine learning techniques using feedback from the users to enable modifiable and self-improving models.

As each of the recording- and data acquisition setups and their belonging classification techniques have inherent strengths and limits (Table 3), a “method-of-choice” for automated GMA does not seem to exist. One might think of an ideal solution that combines multiple setups to complement each other. However, bearing in mind classic GMA’s non-intrusive principle and its merits of being easy-to-use, time- and cost-efficient, to scale up GMA, we must avoid sophisticated, time-consuming, or intrusive setups (e.g., combining wearable sensors or markers with a complex video recording system requiring to configure and calibrate multiple 3D cameras). Such setups are constrained by intricate technical requirements both for data acquisition and processing. For one thing, these setups may influence the infant’s motor pattern, as discussed above. For another, they may prove unsuitable for everyday clinical implementation, being especially inapt in low-resource settings. This way, we would lose the basis for realising the ultimate goal of worldwide routine application of GMA. Nonetheless, depending on the purpose of the respective automated tool, e.g., precise clinical judgement versus initial rapid screening for further referrals or diagnostics, one needs to weigh in on the recording and data acquisition setups and choose and design an appropriate combination.

Regarding current tracking techniques, state-of-the-art methods such as DeepLabCut (Mathis et al., 2018) and OpenPose (Cao, Martinez, Simon, Wei, & Sheikh, 2019) show promising results when tracking both animals and human adults. A new commercial framework, WrnchAI (WrnchAI, 2020) is reported to offer much faster and more accurate adult movement tracking than OpenPose (Gupta, 2020). Whether this holds true for young infants is an open question. As pose estimation includes skeleton constraints as additional *prior* information, it needs to be examined whether such constraints truly improve the movement detection, or whether they might not be permissive for GMA, hence hindering automated detection (Rahmati, Aamo, Stavdahl, Dragon, & Adde, 2014).

Some additional technical considerations may also improve the classification models. For example, having obtained sufficient annotated data, common practice in the field is to split the dataset into three parts, i.e., training set to update model parameters, validation set to evaluate model overfitting, and testing set to assess the classification accuracy and how well the model generalises to new data. If only a small dataset is available, data splitting will become challenging and additional strategies will be required (e.g., Beleites, Neugebauer, Bocklitz, Krafft, & Popp, 2013; Riley et al., 2020; Shahinfar, Meek, & Falzon, 2020). Furthermore, given that a considerable number of features have been extracted and presented by the different studies, whether or not to include a feature pre-selection step is still an open question, depending also on the movement detection (e.g., movement shape vs body pose estimation) and learning (e.g., supervised vs unsupervised) approaches used. Without pre-selection, a significantly higher number of variables must be explored by the classification algorithm. Finally, the most popular algorithms for movement classification are currently SVMs, Random Forests and CNNs, due to their simplicity and straightforward application for a large variety of problems. Novel algorithms have been introduced to the field of automated GMA, such as the Naive Gaussian Bayesian Surprise (NGBS), applied to calculate how much each infant’s movements in a dataset deviate from a group of typically developing infants as the indicator of risk for atypical GMs (Chambers et al., 2019). Similar as in choosing the suitable sensing setups, the selection of the most appropriate algorithm is also contingent on, among others, the data acquisition approaches, the dataset characteristics, and the goal of classification and detection.

Regardless of the technological refinements, currently, automated solutions are developed to complement, but not to replace human assessment in clinical practices. Extending the machine learning technology of tracking and classifying the GMs, future computer-based approaches with multimodal setups (e.g., motion-sensor, pressure-sensitive matt, eye-tracker) may be developed to improve human performance by actively supporting the GMA assessors in real-time across multiple training and clinical settings.

## 5 Conclusion

Automated video-based approaches, being authentic to the non-intrusive principle of the classic GMA, supported by rapid advancements in AI technologies, have the potential to scale up the clinical application of GMA. Technology advancements will enable better data pre-processing (e.g., image enhancement, noise attenuation, region-of-interest detection), improve feature extraction and analyses and lead to an objective and more accurate prediction. Currently, automated GMA models are yet inferior to human experts. Despite their classification performance, current models can deal with but a fraction of the tasks (e.g., some binary or multiple classifications) that a human expert can solve in a standard GMA of a few minutes (e.g., evaluating simultaneously the movement characteristics including complexity and variability, age-specific repertoire, posture, and motor optimality). It is, thus, unlikely that human assessors can be replaced by fully automated systems in the near future. To improve computer-based approaches, there is still a lot to learn from the human GMA experts. This concerns prerequisites for performing GMA and evaluation process embracing manifold aspects to encapsulate infant movements. While developing automated detection and classification models for GMA, a parallel line of research is needed aiming at interactive, real-time support and training for human GMA assessors. By supplementing human faculties (versatile and adaptable to complex and ever-changing situations, proficient in transferring rich experience to novel situations) with computerised tools (objective, stable, fast, and extendable), a future augmented GMA may yield outstanding performance, superior to what humans or computers could achieve alone.

While recent studies focused primarily on the prediction of CP, it is crucial for future research to look beyond this narrow field and open up to the potential of applying GMA to identify deviant early motor functions in infants with various developmental and neurological disorders, infectious diseases affecting the developing nervous system, and genetic disorders. Availing of the advanced computer-vision technology, GMA may be employed to detect more general disintegrity of the developing nervous system through fine-grained high-standard analyses of infant early motor functions. Based on the profound understanding of GMs, incorporating state-of-the-art technology, we are envisioning a worldwide daily clinical application of GMA for the youngest population in the near future.

## Declaration of Competing Interest

We declare that there are no conflicts of interest, guiding this research work.
